# Clinical spectrum of woolly hair: indications for cerebral involvement

**DOI:** 10.1186/s13052-017-0417-1

**Published:** 2017-11-02

**Authors:** Piero Pavone, Raffaele Falsaperla, Massimo Barbagallo, Agata Polizzi, Andrea D. Praticò, Martino Ruggieri

**Affiliations:** 1 University-Hospital “Policlinico-Vittorio Emanuele,” Catania University, Catania, Italy; 2 Unit of Pediatrics and Pediatrics Emergency, Hospital “G. Garibaldi”, Catania, Italy; 3 National Centre for Rare Diseases, Istituto Superiore di Sanità, Rome, Italy; 4 Department of Clinical and Experimental Medicine, Section of Pediatrics and Child Neuropsychiatry, Catania University, Via Santa Sofia 78, 95100 Catania, Italy; 5 Maurice Wohl Clinical Neuroscience Institute, King’s College London, London, UK

**Keywords:** Wooly hair, Localized, Generalized, Rasmussen’s encephalopathy, Epilepsy

## Abstract

**Background:**

Woolly Hair is an uncommon congenital anomaly of the scalp hair presenting with strongly coiled hair involving a localized area of the scalp or covering the entire side and occurring in non-black people. Isolated or localized wooly hair is usually benign and is not related to other disorders and/or complications. On the contrary, the generalized type may be related to disorders and syndromes affecting heart, cutis, liver and gastrointestinal organs. Among the syndromes presenting with wooly hair, the most known are the Naxos syndrome, the Carvajal-Huerta syndrome, the wooly hair/hypotrichosis, the ectodermal dysplasia-skin fragility, the tricho-hepato-enteric syndrome.

**Case presentation:**

To our knowledge, no cases of wooly hair syndromes has been associated to neurologic involvement. Among the clinical notes of patients admitted in the Pediatric Units of the Catania University, we have selected four individuals presenting wooly hair, who showed different clinical features and course: case 1 presenting with a localized wooly hair type; case 2, member of a family affected by WH with autosomal dominant inheritance, not associated to complications; case 3, a wooly hair patient who displayed a progressive, severe form of Rasmussen’s encephalitis with fatal evolution, and case 4, wooly hair associated to brain malformation and drug-resistant epilepsy.

**Conclusions:**

With this report, we aim to underline the wide spectrum of clinical presentation of individuals with WH and in particular we wish to give an annotation on a possible association of WH with severe neurologic disorders.

## Background

Hair disorders in children may be expression of several genetic, cutaneous and systemic disorders. Hair begins to develop between the third and fourth month of fetal life from cylindrical invaginations of the epidermal cells. The hair follicles are organic structures aimed to produce hair [1, 2]. The hair production follows a cyclic sequence of phases: during the growth period, called the “anagen phase”, the follicular cells actively grow, divide and keratinize, while in the “telogen phase” the follicles are in a rest-stage and hair growth temporarily ceases. “Catagen phase” expresses a transitional period between the anagen and telogen phases. In human beings, the anagen phase for each scalp hair may vary from two to 5 years, followed by telogen phase lasting three to 6 months. Hair anomalies, often involving the hair shaft, may present with changes in color, density, length and structure [3] and may have a congenital or acquired origin.

**Table 1:** The “Woolly hair spectrum”

			Clinical features	Genes
Localized			WH isolated (patchy)	
			WH nevus + epidermal nevi	*HRAS* (15)
Generalized	Syndromic		WH-PPK-ARVC (Naxos disease) ARCV + mild WH and PPK	*JUP* (16) *DSC2* (17)
			WH-PPK-LDC (Carvajal-Huerta syndrome)	*DSP* (18)
			WH-PPK-pseudoainhum leuconychia	*KANK2* (12)
			WH-ectodermal dysplasia-skin fragility	*PKP1* (13)
			WH-diarrhea-inflammatory skin lesions	*ADAM17* (19)
			WH-diarrhea-facial dysmorphism-immunodysfunction (tricho-hepato-enteric syndrome)	*TTC37* (20) *SKIV2L* (21)
			WH-anomalous pigmentation-unilateral cerebral involvement	
			WH-cerebral ectopia-polymicrogyria	
	Non syndromic	AR	WH-hair loss	
		AD	WH-hypotrichosis (mild and severe)	*KRT71* (22) *KRT74* (23)

*ADAM17* metallopeptidase domain 17 gene; *ARVC* Arrhythmogenic right ventricular cardiomyopathy; *DSC2* desmosomal cadherin gene; *DSP* desmosomal plaque gene; *HRAS* Harvey Rat Sarcoma Viral Oncogene Homolog gene; *JUP* junction plakoglobin gene; *LDC* Left dilated cardiomyopathy; *KANK2* KN Motif And Ankyrin Repeat Domains 2 gene; *KRT71* Keratin 71 gene; *KRT74* Keratin 74 gene; *PKP1* plakophilin gene; *PPK* palmoplantar keratoderma; *SKIV2L* superkiller viralicidic activity 2-like gene; *TTC37* Tetratricopeptide repeat-containing 37 gene; *WH* Woolly hair

Woolly Hair (WH) is an uncommon congenital abnormality of the scalp hair consisting, by definition, of strongly coiled hair localized in a side or totally involving the scalp occurring in non-black people [4, 5].

The term was first introduced by Gossage [6] in a European family presenting this sign with an autosomal dominant inheritance. A first classification was reported by Hutchinson et al. [7], who distinguished the WH in three sub-types: the isolated or localized type, the hereditary dominant and the familial recessive. Successively the generalized type was distinct in syndromic and non-syndromic and the last one in autosomal dominant and autosomal recessive [5–8].

Recently we have reviewed the clinical notes of patients admitted to University-Hospital “Policlinico-Vittorio Emanuele” Catania University, Italy, and selected 4 individuals presenting WH, a phenotype and a course each different from the others. We report here: a) a case of localized WH; b) a girl and her family with autosomal dominant WH without associated complications; c) a patient with WH who developed Rasmussen’s encephalitis with fatal evolution; d) a patient with WH who manifested severe drug-resistant epileptic seizures linked to cerebral malformation. With this report, we wish to underline the wide clinical spectrum of individuals with WH, drawing the attention, in particular, on the possible association of WH with cerebral involvement, an issue that has not been previously reported.

## Case presentation

### Case 1

A one-year-old boy. No family history of cutaneous or hair anomalies were reported. The mother denied having had infectious disease during her pregnancy nor having taken drugs or alcohol. Birth-weight, length and head circumference were within normal limits. The hair’s anomaly had been present since the first months of life. When he came to our observation, the scalp hair presented with a circumscribed patch of kinky, curly, lighter hair occupying the temporo-parieto-occipital area of right side. The anomalous hairs were difficult to comb but not fragile. The boy has been followed-up for 3 years. No abnormalities on physical examination nor in the routine laboratory findings were found.

### Case 2

A five-year-old girl came to our observation for bronchitis [9, 10]. At the physical examination the girl showed, beside the signs of a respiratory infection, a typical form of generalized short curlier, woolly hair covering all the scalp. On microscopic examination, the hair appeared to be thinner than normal for age and to be twisted on the longitudinal axis. The family history disclosed the presence of this hair anomaly in 23 members of the family, inherited as an autosomal dominant trait with incomplete penetrance, as in some of the family members the hair presented with a intermediate feature between normal and anomalous hair [9, 10].

We have recently visited the girl in her adulthood: she referred to have, neither her or her family, complained disturbances related to WH.

### Case 3

This 29-month-old girl was the third child of healthy unrelated Italian parents. She was born at 36 weeks of gestation, by planned cesarean section, after a pregnancy complicated by maternal gestosis. At birth, her weight was 2340 g, her height 49 cm and head circumference 35 cm. The family history was negative for skin, hair and systemic disorders. The mother denied to have had infectious episodes during her pregnancy or to have used drugs or alcohol. Fetal ultrasound examination had not identified growth anomalies or other abnormalities. Fetal movements had been normally felt. The hair anomaly was noticed since birth. Her psychomotor development was referred as normal. The child was first admitted to the Pediatric Department of Catania University at the age of 25 months for consultation due to a second episode of generalized tonic-clonic seizures of 10 min. of duration, solved after an intravenous infusion of benzodiazepine. The previous convulsive episode had been registered a week before.

**Fig. 1:**
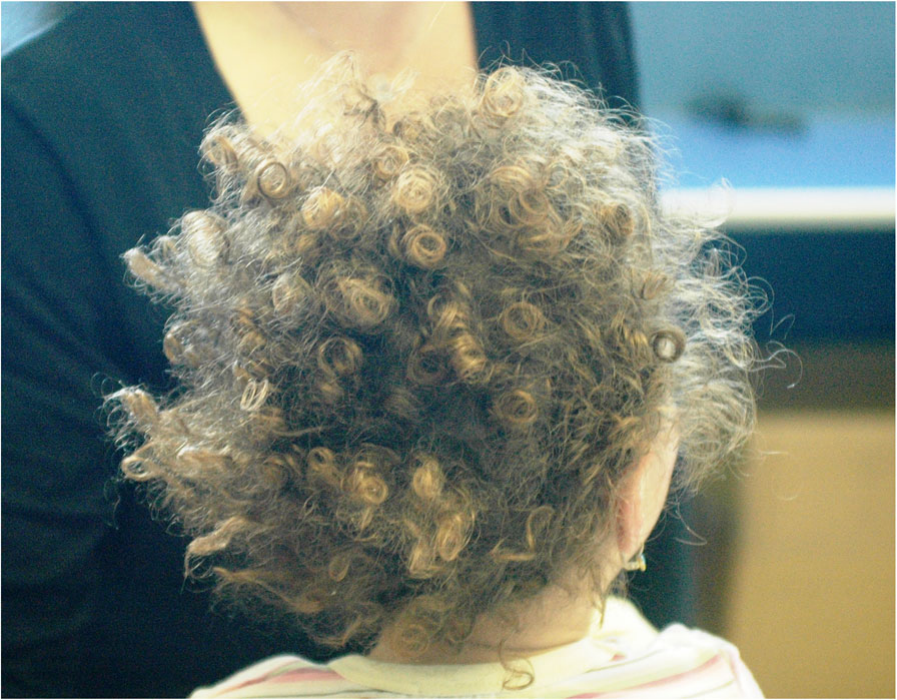
Patient 3, at 2.5 years of age. Woolly hair localized all over the scalp

**Fig. 2:**
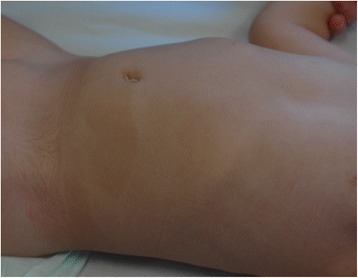
Patient 3, at 2.5 years of age. The presence of hyperpigmented areas in some areas of the abdomen

**Fig. 3:**
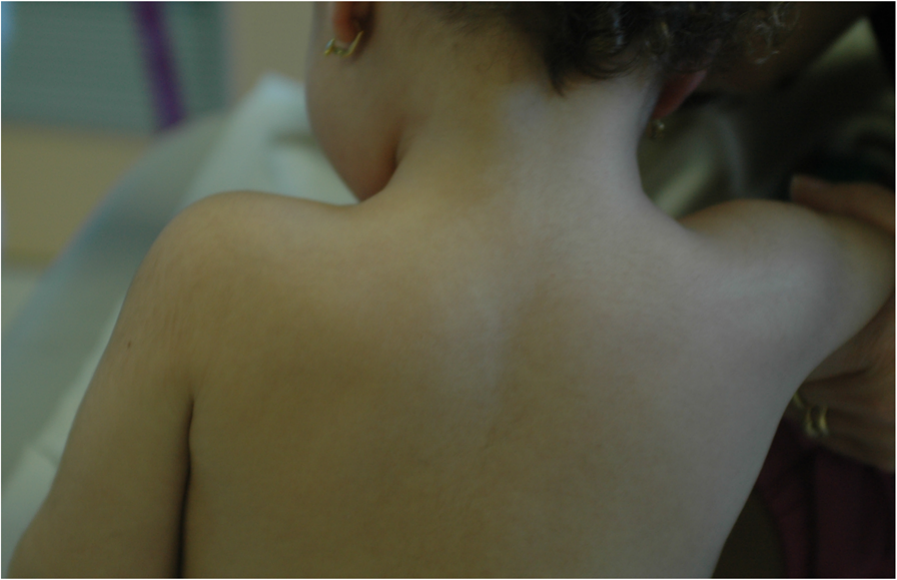
Patient 3, at 2.5 years of age. An area of hypopigmentation can be seen in the upper trunk

At the physical examination, the girl was in good general condition. Her weight was 13 kg (between 50^th^ and 75^th^percentile), length 89 cm (90^th^ percentile) and head circumference 47 cm (between 25^th^ and 50^th^ percentile). The hair, in all the scalp, were strongly curled, woolly, lighter in color (Fig. 1) compared to those of her brothers. The diameter of the hair was 0.5 mm. Oral mucosa was of normal colour. In several body areas, cafè-au-lait spots were noticed and involved (Fig. 2): left iliac fossa (7 × 3 cm), left hip (4 × 2 cm), right hypocondrium (3 × 2 cm), left gluteus (0.5 × 0.5 cm). Hypopigmented spots were also noticed in-between the shoulder (4 × 4 cm) (Fig. 3). Ear, heart, respiratory and abdominal organs were normal as were the neurologic examination. The stages of psychomotor development including language were initially appropriate for the age. Routine laboratory analyses, including urinary organic acids, plasmatic aminoacids, were normal as ECG and echocardiogram. The EEG showed a slight asymmetric background with low rhythm of middle voltage in the right hemisphere. Spike/waves complexes, synchronic and asynchronic were registered in the central and temporal areas of both the hemispheres, with a dominance in the right side. Treatment with valproate was started at a dosage of 20 mg/Kg. Brain MRI, skeletal X-rays and other laboratory findings were normal. A month later the girl came back to our observation for new episodes of generalized tonic-clonic seizures with a frequency of 1–2 for week. Treatment with topiramate was gradually added to the valproate. After a short period of bettering, 2 months after, a new admission to the hospital was required due to an increase of epileptic seizures, mainly localized in the left side. These episodes were recurrent. At physical examination, the sensorium appeared clouded, and her Glasgow Coma Score was 12. The seizures were of clonic-tonic type, localized in left side of the body and complicated by hemiplegic episodes. Some of the epileptic episodes were complicated by status epilepticus. In the interictal phase the walk was slightly ataxic. The EEG showed asymmetric background and presence of slow middle voltage rhythms on both areas with generalized spike-waves complexes prevalent in the centro-temporal right area. Lumbar puncture disclosed a limpid liquor with normal pressure and no cellular increase. A new brain MRI showed a diffuse area of hyperechogenicity, more evident in the long TR sequences, localized in cortico-subcortical areas of the right temporo-parietal lobes. Treatment with acyclovir, immunoglobulins and cortisone was ineffective. Diagnosis of Rasmussen’s encephalitis was made. After a short period of bettering there was a worsening of the disorder, which caused the death of the patient.

### Case 4

**Fig. 4:**
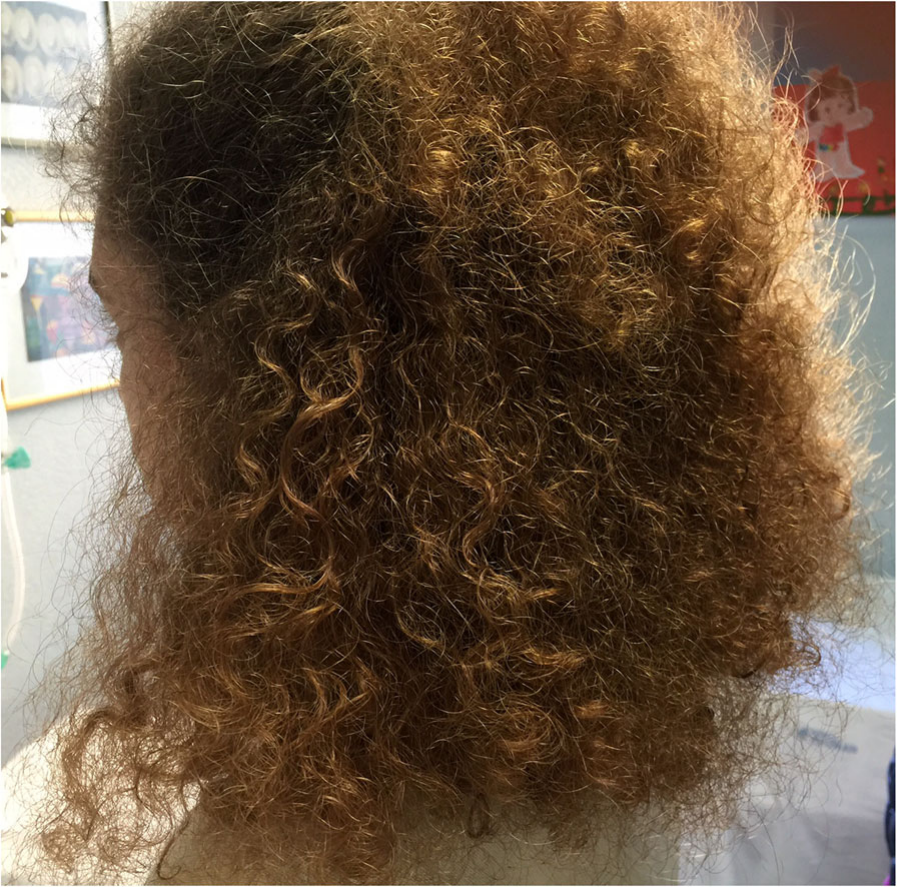
Patient 4, at 12 years of age. Woolly hair covering all the scalp

**Fig. 5:**
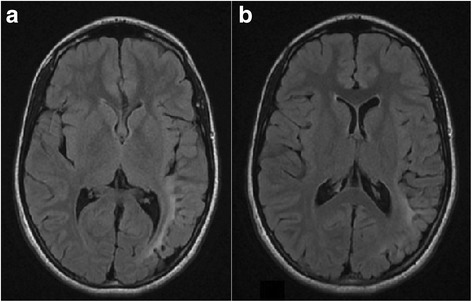
**a**, **b** Patient 4, brain magnetic resonance imaging, axial T2 FLAIR scans. An area of altered signal of the white matter in the temporal and parietal lobes can be observed, together with a reduction of the gray matter thickness in the left occipital sulcus (polymicrogiria)

This 12-years-old girl was born at term after a normal delivery. Family history was irrelevant for cutaneous and systemic anomalies. The mother denied to have taken drugs or to have had infectious diseases during her gestation. Both parents were healthy and non-consanguineous. Birth-weight was 2800 g, height 50 cm and head circumference 35 cm. Psychomotor development was normal: she started walking at the age of 14 months and pronounced her first words at the age of 13 months. Since during the first months of life she presented with strongly coiled hair, difficult to comb, localized in the entire scalp. Psychomotor development was normal. At the age of 7 years she had the first convulsive episode during sleep consisting in focal tonic-clonic seizures with subsequent generalization. An EEG showed the presence of spikes and waves complexes prevalent in the left temporal areas. A brain MRI disclosed abnormalities involving the left temporal lobe. Although treatment was started with valproate, the epileptic seizures continued with increase in frequency and duration. The school performance was poor and the behaviour of the girl was progressively more aggressive and violent. After a brief period of slight improvement, the epileptic seizures continued in spite of the treatment with other anticonvulsants in add-on. A diagnosis of drug-resistant multifocal epilepsy, prevalently affecting the left hemisphere, was made. The epileptic seizures were associated to other neurologic signs such as sensory-motors manifestations, scream when touched and frequently throw tantrums. Aside the hair structure (Fig. 4), the girl did not show other abnormal malformations. Laboratory findings, including urinary organic acids and plasmatic aminoacids, were normal as were ECG and heart ultrasound. After 6 months, a new EEG showed multifocal spike-waves and the cerebral MRI disclosed the presence of left occipito-temporal polymicrogyria (Fig. 5a, b). Epileptic surgery treatment was not advised. Treatment with carbamazepine and clobazam gave poor results and the girl continued to present very frequent seizures.

## Discussion

In the non-black races, WH is certainly an uncommon anomaly of the hair structure with a wide range of clinical expression ranging from simple cosmetic issues to more or less severe associated disorders. The cases here reported represent a clear example of the heterogeneity of this topic. We have collected four sample-cases of WH in a long time, thus demonstrating the sporadic event of this anomaly and in two of them, a severe cerebral involvement was found.

In case 1, the WH was localized consisting with the diagnosis of benign WH. Aside the curled, woolly hair, no associated disorders were reported at clinical and laboratory examinations. The family and the boy have psychologically accepted the scalp hair anomaly without particular problems. In case 2, the WH was generalized, involving the entire scalp, and was reported to be present in several members of the family through an AD inheritance with incomplete penetrance. In a recent interview carried out to the patient, now in her adult age, no particular or specific disorders related to the hair condition were referred regarding herself and the family.

Inversely from the cases upper-mentioned, the other two patients showed particularly severe neurologic disorders as the most relevant clinical signs, which in our statistics have not been reported in patients with WH. One, beside WH, showed a complex pattern of cutaneous macules, both hypo- and hyperpigmented, in different areas of the body, progressive severe epileptic seizures suggestive of Rasmussen’s encephalitis, that resulted in a fatal evolution. The proband 4 showed drug-resistant epileptic seizures, severe behavioural disturbances and a complex brain malformation with signs of polymicrogyria.

Localized WH is a usually benign condition rarely associated to other disorders. The generalized type, on the contrary, has been observed in patients with associated anomalies including cardiomyopathy, cutaneous abnormalities (i.e. palmoplantar keratoderma or epidermal nevus) and other disorders encompassing the diagnosis of well-known syndromes.

Recent advances in genomic analyses have showed that events involving keratin, adherens junctions and signal transduction proteins are at the basis of the WH anomaly. Several genes codifying desmosomal proteins, keratin, lipid mediators and other components have been recognized to cause syndromic and non syndromic WH [5].

The most known syndromes that manifest with WH as one of the presenting signs are the Naxos syndrome, the Carvajal-Huerta syndrome, the WH/hypotrichosis, the ectodermal dysplasia-skin fragility and the tricho-hepato-enteric syndrome. Naxos syndrome is an autosomal recessive disorder characterized by the triad arrhythmogenic right ventricular cardiomyopathy, WH and palmoplantar keratoderma. In this disorder, a lifelong follow-up with serial cardiac assessments and the prevention of progression of the hearth disturbances are essential to avoid heart failure which can lead to a sudden death [5, 11]. Features similar to those of Naxos syndrome are reported in the Carvajal-Huerta syndrome, in which the palmoplantar keratoderma and WH are associated to dilated cardiomyopathy of juvenile onset, involving particularly the left ventricle and causing early morbidity [12]. Two families with members affected by keratoderma, WH, leukonychia, pseudoainhum of the fifth toe without cardiac involvement were reported by Ramot et al. [13]. Ectodermal dysplasia-skin fragility syndrome (ED-SFS) is an autosomal recessive genodermatosis: in one of the reported patients, a 14-month-old infant, beside the ED-SFS, others anomalies such as scalp WH, sparse eyelashes and eyebrows, abnormal dental development and a desquamating erythematous rash were also observed [14]. WH may be also a sign of tricho-hepato-enteric syndrome, which manifests with severe infantile diarrhea, failure to thrive, dysmorphism, immune or hepatic dysfunction and trichorrhexis nodosa [15]. In the table, the main disorders associated with WH and the causative genes are reported (Table 1) [16–24].

As mentioned above, the clinical manifestations associated to WH are quite wide and involve a large spectrum of body organs. The heart involvement is known since the 1986, by the first report of Protonotarios et al. [25].

Neurologic involvement, observed in two of the present patients, has never been reported in WH, but is frequent in some mosaic neurocutaneous disorders presenting with hairlessness or alopecia (e.g. encelphalocraniocutaneous lipomatosis and incontinentia pigmenti) [26]. Patient 3 showed, beside the WH, an anomalous pattern of congenital cutaneous manifestations with areas of hypo- and hyperpigmented lesions and a progressive neurologic worsening lasting 4 months with severe drug-resistant epileptic seizures resulting in death. A diagnosis of Rasmussen’s encephalitis, a rare inflammatory neurologic disorder, mainly affecting a single cerebral hemisphere was made. The other (patient 4) suffered from a severe cerebral malformation with intractable epileptic seizures and a relevant aggressive and irritable behavioural.

## Conclusions

A pathogenetic event affecting the common neuroectodermal origin of hair, cutis and brain may be at the basis of the anomalies present in the probands with a congenital predisposition to severe inflammatory processes for the patient 3 and a congenital structural cerebral malformative impairment for the patient 4. More similar observations as those here reported are required to include WH associated to neurologic symptoms among the other neurocutaneous syndromes. Unfortunately no molecular assessment was performed in the patients 3 and 4, due to the refusal by the parents and in the case of Rasmussen’s encephalitis for the rapid and unexpected outcome of the patient. The cases here reported may extend the spectrum of the WH, pointing out a possible association with brain disorders.

## Abbreviations

AD: Autosomal dominant
ECG: Electrocardiography
ED-SFS: Ectodermal dysplasia-skin fragility syndrome
EEG: Electroncephalography
MRI: Magnetic Resonance imaging
WH: Wooly Hair
